# Supplementary material for: A child with perinatal HIV infection and long-term sustained virological control following antiretroviral treatment cessation
Source: Nat Commun. 2019 Jan 24;10:412. doi: 10.1038/s41467-019-08311-0 (PMC6345921; doi:10.1038/s41467-019-08311-0)
Supplement: Supplementary file 1 — Supplementary Information [file 41467_2019_8311_MOESM1_ESM.pdf]

Supplementary information

A child with perinatal HIV infection and long-term sustained virological control following antiretroviral treatment cessation

Violari *et al*.

Supplementary Figures

SEQ child: 1372 bp;
Composition 506 A; 274 C; 324 G; 268 T; 0 OTHER
Percentage: 37% A; 20% C; 24% G; 20% T; 0%OTHER

```
Molecular Weight (kDa): ssDNA: 425.63    dsDNA: 845.8
ORIGIN
903    CAGGGAGCTG GAAAGATTTG CACTTAACCC TGGTCTTTTA GAGACACCAG AAGGCTGTAG
963    ACAAATAATG ACACAGCTAC AACCAGCTCT TCAGACAGGA ACAGAGGAAC TTAAATCATT
1023   ATTTAACACA GTAGCAACGC TCTATTGTGT ACATGCACAG ATAGAGGTAC GAGACACCAA
1083   GGAAGCCTTA GACAGGATAG AGGAAGAACA AAGCAAAAGT CAGCAAAAAG CACAGCAAGC
1143   AGAAATGGCT GATAGAGGAA AGATCAGTCA AAATTATCCT ATAGTGCAGA ATATTCAAGG
1203   GCAAATGGTA CACCAGCCCA TATCACCAAG AACTTTGAAT GCATGGGTAA AAGTAATAGA
1263   GGAGAAGGCC TTCAGCCCAG AAGTGATACC CATGTTTACA GCATTATCAG AAGGAGCCAC
1323   CCCACAAGAT TTAAACACCA TGTTAAATAC AATAGGGGGA CATCAAGCAG CCATGCAAAT
1383   GTTAAAAGAT ACCATTAATG AGGAAGCTGC AGAATGGGAT AGGATACATC CAGTACATGC
1443   TGGACCTGTT GCACCAGGCC AAATGAGAGA ACCAAGGGGA AGTGACATAG CAGGAACTAC
1503   TAGTACCCTT CAAGAACAAA TAGCATGGAT AACAGGTAAC CCACCTATTC CAGTAGGAGA
1563   CATCTATAAG AGATGGATAA TTCTGGGGTT AAATAAAATA GTAAGAATGT ATAGCCCTGT
1623   CAGCATTTTG GACATAAGAC AAGGGCCAAA AGAACCTTTT AGAGACTATG TAGATCGGTT
1683   CTTTAAAACT TTAAGAGCTG AACAAGCTAC ACAAGAAGTA AAAAATTGGA TGACAGACAC
1743   ATTGTTAGTC CAAAATGCAA ACCCAGATTG CAAGACCATT TTAAGAGCAT TAGGACCAGG
1803   GGCTACATTA GAAGAAATGA TGACAGCATG TCAGGGAGTG GGAGGACCTA GCCACAAAGC
1863   AAGAGTTTTG GCTGAGGCAA TGAGTCAAGC AGGCAGTGCA GGCATAATGA TGCAGAGAAG
1923   CAATTTTAAA GGCCCTAGAA AAATTGTTAA ATGTTTCAAC TGTGGCAAGG AAGGGCACAT
1983   AGCTAGGAAT TGCAAGGCTC CCAGGAAAAA AGGCTGTTGG AAATGTGGAA GGGAAGGACA
2043   CCAAATGAAA GACTGCACTG AAAGGCAGGC TAATTTTTTA GGGAAAATTT GGCCTTCCCT
2103   CAAGGGGAGG CCAGGGAATT TCCTTCAGAG CAGACCAGAA CCAACAGCCC CACCAGCAGA
2163   GAGCTTCAGG TTCGAGGAGA CAACCCCCAC TCCGAAACAG GAGTCGAGAG ACAAGGAACC
2223   ACCCTTAACT GCCCTCAAAT CACTCTTTGG CAGCGACCCC TTGTTACAAT AA
```

Supplementary Figure 1. *Consensus partial gag-pol sequence for the Case.* A consensus HIV-1 proviral partial *gag-pol* nucleotide sequence (HXB2 numbering nt 903-2292) was derived from alignment of 20 cloned sequences for the child (Genbank accession numbers MH789553-MH789572). Sequence denotes partial *gag* (903-2292) and partial *pol* (nt 2085-2292). Further breakdown of coding sequences includes partial *gag* p17 (nt 903-1185, aa 39-132), *gag* p24 (nt 1186-1878, aa 1-231), *gag* p2 1879-1920, aa 1-14), *gag* p7 (nt 1921-2085, aa 1-55), *gag* p1 (nt 2086-2133, aa 1-16), *gag* p6 (nt 2134-2292, aa 1-53) and partial protease sequence (nt 2253-2292, aa 1-14). Numbering according to the Los Alamos HIV Sequence database, www.hiv.lanl.gov

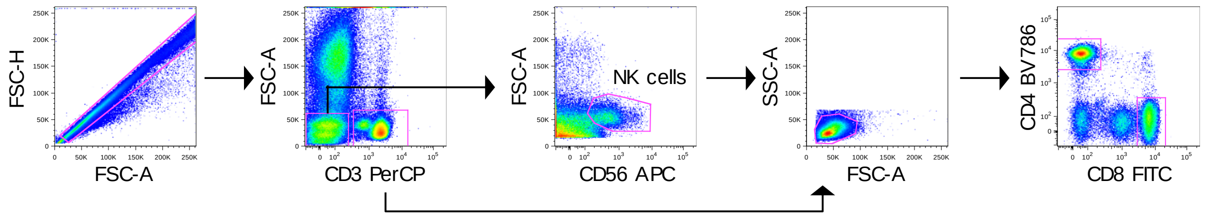

Supplementary Figure 2. *Gating strategy for the identification of CD4+ and CD8+ T-cells and NK cells for the intracellular cytokine assay (manuscript Fig. 3c, d).* Singlets were identified using FSC-H against FSC-A followed by gating on CD3+ and CD3- cells. CD3+ T-cells were further gated on low SSC-A vs low FSC-A (small lymphocytes) and then on CD4+ and CD8+ T-cells. NK cells were identified by gating CD3- CD56+ cells.
Antibody staining panel: CD3 PerCP, CD4 BV786, CD8 FITC, CD56 APC, IFN-γ PE, IL-2 PE.

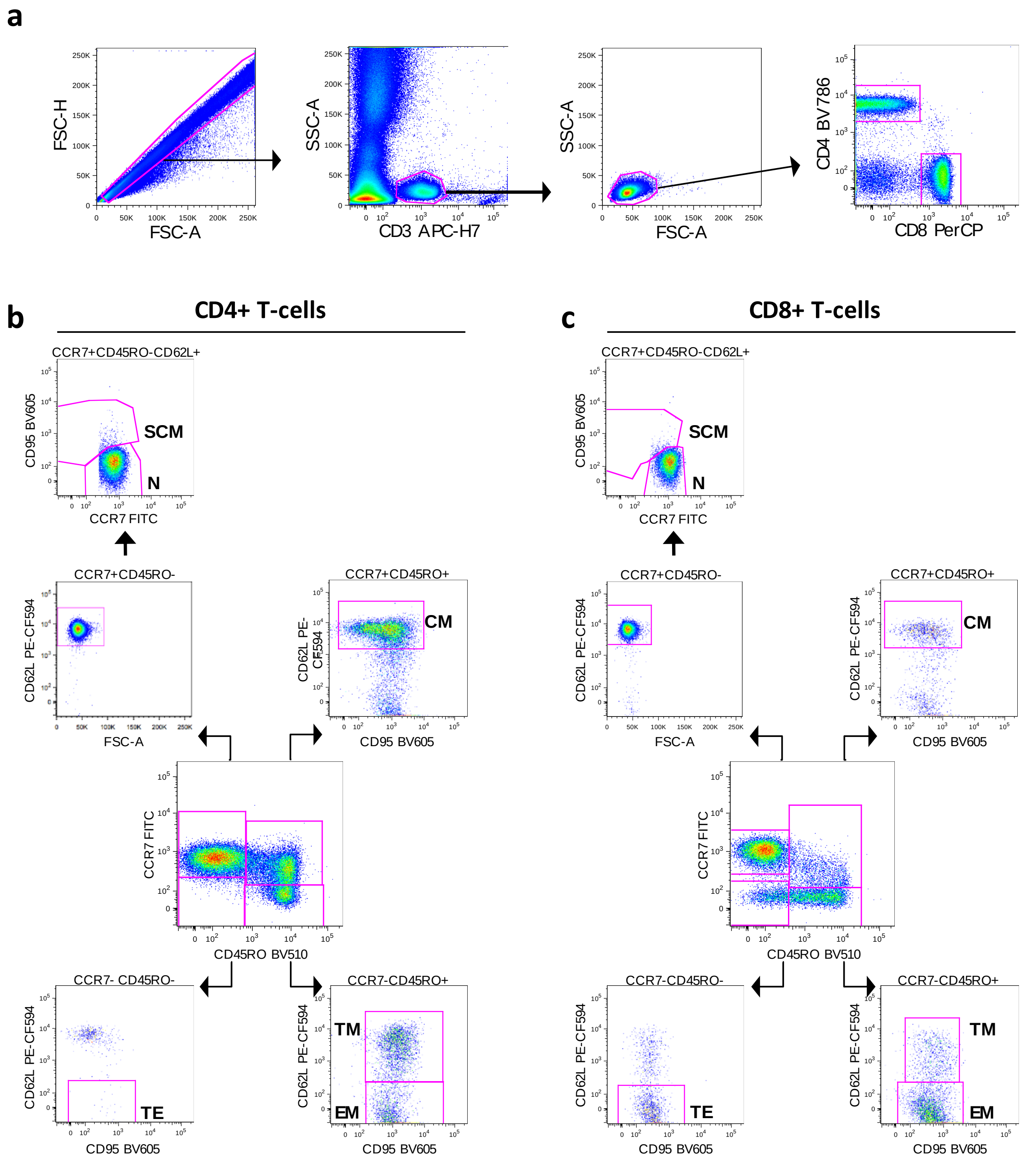

Supplementary Figure 3. *Gating strategy used to immunophenotype memory T-cell subsets (manuscript Fig.5b)*. Representative flow cytometry plots are from the Case. **a** Singlets were identified followed by gating on CD3+ lymphocytes. CD3+ T-cells were further gated on low SSC-A and FSC-A (small lymphocytes) and then on CD4+ or CD8+ T-cells. **b** Gating strategy for the determination of CD4+T-cell subsets: Naïve (N): CCR7+CD45RO-CD95-; Stem cell Memory (SCM): CCR7+CD45RO-CD95+; Central Memory (CM): CCR7+CD45RO+CD62L+; Transitional Memory (TM): CCR7-CD45RO+CD62L+; Effector Memory (EM): CCR7-CD45RO+CD62L- and Terminal Effector (TE): CCR7-CD45RO-CD62L-. **c** Gating strategy for determination of CD8+ T-cell subsets is similar to that for CD4+ T-cells (**b**). For the low frequency T_{SCM} subset, the gating strategy outlined by Lugli *et al*[1] was utilized: the slightly lower level of CCR7 expression on T_{CSM} compared to T_N was utilized to provide a clear separation of positive and negative CD95 cells. A diagonal gate was drawn on the bivariate plot of CD95 versus CCR7 on the total T cell population (not shown) and then copied into the same bivariate plot after gating for CD45RO, CCR7 and CD62L.

Antibody staining panel: CD3 APC-H7, CD4 BV786, CD8 PerCP, CD45RO BV510, CCR7 (CD197) FITC, CD95 BV605, CD62L PE-CF594.
Immunophenotyping data (Supplementary Table 3).

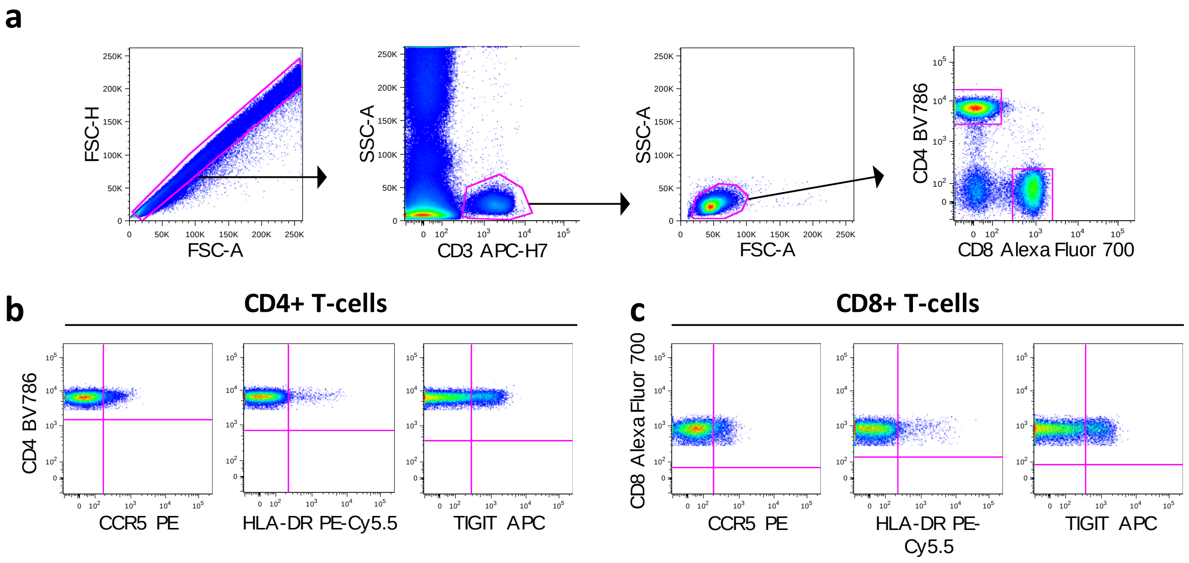

Supplementary Figure 4. *Gating strategy for determining CCR5, HLA-DR and TIGIT expression on CD4+ and CD8+ T-cells (manuscript Fig. 5c)*. **a** Singlets were identified followed by gating on CD3+ lymphocytes. CD3+ T-cells were further gated on low SSC-A and FSC-A (small lymphocytes) and then on CD4+ or CD8+ T-cells. The expression of CCR5, HLA-DR and TIGIT on (**b**) CD4+ T-cells and (**c**) CD8+ T-cells are shown for the Case, respectively. Antibody staining panel: CD3 APC-H7, CD4 BV786, CD8 Alexa Fluor® 700, CCR5 PE, HLA-DR PE-Cy5.5, TIGIT APC.

Immunophenotyping data (Supplementary Table 3).

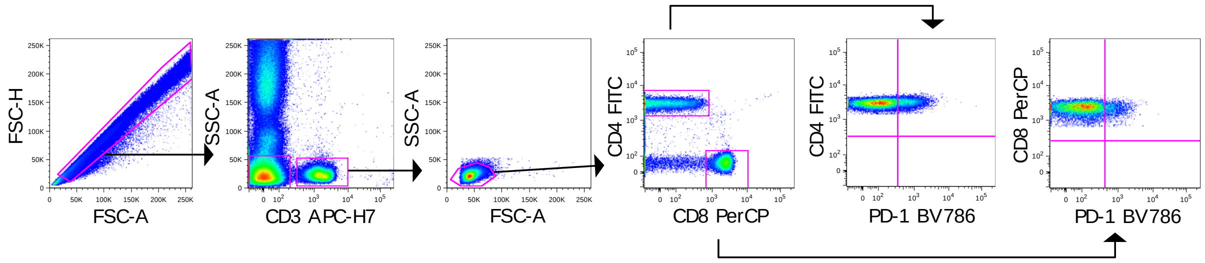

Supplementary Figure 5. *Gating strategy for determining PD-1 expression on CD4+ and CD8+ T-cells (manuscript Fig.5c)*. Singlets were identified followed by gating on CD3+ lymphocytes. CD3+ T-cells were further gated on low SSC-A and FSC-A (small lymphocytes) and then on CD4+ or CD8+ T-cells. PD-1 expressing CD4+ and CD8+ T-cells are shown for the Case.

Antibody staining panel: CD3 APC-H7, CD4 FITC, CD8 PerCP, CD279 (PD-1) BV786. Immunophenotyping data (Supplementary Table 3).

Supplementary Tables

Supplementary Table 1. Longitudinal viral loads and CD4 T-cell counts and percentages of the Case (Fig.1a-c).

Phase	Study Week	Age (weeks)	Age (years)	HAART	CD4 count	CD4 percentage	Viral Load	Log VL
Screening	-4.1	5.57	0.11	No HAART	3656	34.1	>750, 000	5.88
Screening	-1.1	8.57	0.16	No HAART	3708	41	151, 000	5.18
Baseline	0.0	9.71	0.19	AZT+3TC+LPV/r	2249	41.6	not done	not done
Primary	4.0	13.71	0.26	AZT+3TC+LPV/r	3583	33.9	915	2.96
Primary	12.0	21.71	0.42	AZT+3TC+LPV/r	2644	46.1	335	2.53
Primary	24.0	33.71	0.65	AZT+3TC+LPV/r	1976	42.8	<50	1.70
Primary	32.0	41.71	0.80	AZT+3TC+LPV/r	2867	43.1	20	1.30
Primary/Interruption Date	40.0	49.71	0.95	AZT+3TC+LPV/r	1968	40.9	<20	1.30
Interruption	48.0	57.71	1.11	No HAART	1857	42.7	<20	1.30
Interruption	60.0	69.71	1.34	No HAART	2543	41	<20	1.30
Interruption	72.4	82.14	1.57	No HAART	2143	38.1	<20	1.30
Interruption	84.0	93.71	1.80	No HAART	1935	47	<20	1.30
Interruption	96.0	105.71	2.03	No HAART	1492	40.4	<400	1.30
Interruption	107.0	116.71	2.24	No HAART	1618	45.9	<400	1.30
Interruption	116.0	125.71	2.41	No HAART	1390	43.8	<20	1.30
Interruption	128.0	137.71	2.64	No HAART	1551	40.1	<20	1.30
Interruption	139.7	149.43	2.86	No HAART	1447	38	<20	1.30
Interruption	152.0	161.71	3.10	No HAART	1349	46.6	not done	not done
Interruption	162.7	172.43	3.30	No HAART	1440	40.8	<20	1.30
Interruption	176.0	185.71	3.56	No HAART	1131	30.8	<20	1.30
Interruption/Final CHER Visit	188.4	198.14	3.80	No HAART	1058	32.93	<20	1.30
Interruption/Post CHER Visit	200.0	209.71	4.02	No HAART	1210	39.5	not done	not done
Interruption/Post CHER Visit	212.0	221.71	4.25	No HAART	1315	38.9	not done	not done
Interruption/Post CHER Visit	236.0	245.71	4.71	No HAART	1234	38.9	not done	not done
Interruption/Post CHER Visit	265.4	275.14	5.27	No HAART	1578	41.9	not done	not done
Interruption/Post CHER Visit	289.6	299.29	5.74	No HAART	1700	44.6	not done	not done
Interruption/Post CHER Visit	321.7	331.43	6.35	No HAART	1080	39.6	<20	1.30
Interruption/Post CHER Visit	353.0	362.71	6.95	No HAART	1028	35.1	<20	1.30
Interruption/Post CHER Visit	354.4	364.14	6.98	No HAART	1028	35.1	<20	1.30
Interruption/Post CHER Visit	379.4	389.14	7.46	No HAART	958	39.6	<20	1.30
Interruption/Post CHER Visit	404.7	414.43	7.94	No HAART	734	39.6	<20	1.30
Interruption/Post CHER Visit	425.0	434.71	8.33	No HAART	918	38.1	<20	1.30
Interruption/Post CHER Visit	430.4	440.14	8.44	No HAART	810	41.2	<20	1.30
Interruption/Post CHER Visit	430.4	440.14	8.44	No HAART	810	41.2	<20	1.30
Interruption/Post CHER Visit	457.4	467.14	8.95	No HAART	975	30.1	<20	1.30
Interruption/Post CHER Visit	484.9	494.57	9.48	No HAART	892	38.6	<20	1.30

Supplementary Table 2. Baseline CD4 counts and CD4 percentages of 227 early treated children who stopped ART in the CHER trial.

No.	Randomisation date	CD4 count	CD4 percentage
1	29Jul2005	1411	45.4
2	04Aug2005	1292	34.8
3	19Aug2005	2122	38.4
4	25Aug2005	2192	29.6
5	07Sep2005	2255	36.8
6	15Sep2005	1981	35.6
7	15Sep2005	2554	41.2
8	26Sep2005	2645	39.1
9	26Sep2005	3071	45.1
10	28Sep2005	2610	39.3
11	11Oct2005	2142	33.3
12	12Oct2005	1804	43.8
13	17Oct2005	2117	28.6
14	17Oct2005	1482	35.8
15	02Nov2005	1772	32.5
16	08Nov2005	2033	33
17	02Dec2005	2436	40.1
18	08Dec2005	3376	45.7
19	14Dec2005	2806	26.5
20	21Dec2005	3086	37.6
21	21Dec2005	2416	37.7
22	04Jan2006	2600	30.9
23	23Jan2006	1863	28
24	25Jan2006	1108	31.1
25	25Jan2006	2012	36.3
26	25Jan2006	2917	40.2
27	03Feb2006	1923	31.5
28	03Feb2006	1739	34.6
29	16Feb2006	2339	40.6
30	17Feb2006	1368	27.6
31	23Feb2006	2763	38.9
32	24Feb2006	2361	34
33	24Feb2006	1543	32.4
34	27Feb2006	2047	42.4
35	27Feb2006	1705	33
36	03Mar2006	2370	42.2
37	08Mar2006	3340	32.4
38	09Mar2006	4258	44.4
39	13Mar2006	1488	35.1
40	17Mar2006	3292	43.4

41	17Mar2006	2387	28.1
42	24Mar2006	2643	36.1
43	27Mar2006	3900	28.9
44	05Apr2006	3403	39.1
45	10Apr2006	2625	48.7
46	10Apr2006	1772	31.1
47	10Apr2006	1822	37.5
48	12Apr2006	4561	35.3
49	19Apr2006	2201	25.3
50	20Apr2006	1763	26.9
51	20Apr2006	3773	33.8
52	25Apr2006	2292	31.4
53	11May2006	2477	39.1
54	15May2006	1075	36.4
55	30May2006	3439	41.7
56	30May2006	1530	28.5
57	31May2006	820	30.2
58	05Jun2006	4368	36.4
59	06Jun2006	2457	37
60	07Jun2006	3085	37.6
61	20Jun2006	2012	42.3
62	22Jun2006	2334	37.8
63	22Jun2006	2210	59.4
64	26Jun2006	2923	43.8
65	27Jun2006	1817	34.2
66	03Jul2006	3185	28.7
67	03Jul2006	2948	26.8
68	04Jul2006	3369	37.4
69	05Jul2006	3729	38.6
70	06Jul2006	2924	36.3
71	10Jul2006	2722	42.2
72	12Jul2006	2098	25.2
73	12Jul2006	5347	52.6
74	19Jul2006	1309	33.1
75	21Jul2006	2378	38.2
76	21Jul2006	950	29.1
77	24Jul2006	3308	41.9
78	24Jul2006	3841	54.5
79	25Jul2006	2982	40.1
80	27Jul2006	1290	32.7
81	31Jul2006	2480	38.6
82	08Aug2006	2662	27.3
83	10Aug2006	2447	39.7
84	10Aug2006	1793	36.1
85	11Aug2006	2212	29.5

86	17Aug2006	1759	28.8
87	21Aug2006	2810	49.9
88	23Aug2006	2136	35.9
89	24Aug2006	2283	36.4
90	25Aug2006	2211	35.1
91	29Aug2006	4572	40.9
92	30Aug2006	4828	41.8
93	31Aug2006	3707	45.7
94	04Sep2006	1125	25.4
95	05Sep2006	2433	36
96	06Sep2006	2351	31.8
97	13Sep2006	2765	42.6
98	15Sep2006	3432	37.2
99	15Sep2006	2017	31
100	21Sep2006	1873	32.4
101	21Sep2006	3017	42.8
102	22Sep2006	1609	32.2
103	26Sep2006	2198	53
104	27Sep2006	1708	28.1
105	27Sep2006	5717	44.7
106	29Sep2006	1962	36.6
107	09Oct2006	1502	25.1
108	11Oct2006	1867	34.4
109	11Oct2006	3695	49.4
110	18Oct2006	2461	28.7
111	23Oct2006	1552	33.6
112	23Oct2006	1213	27.2
113	25Oct2006	3270	38.7
114	31Oct2006	1564	27.8
115	31Oct2006	3091	31.7
116	06Nov2006	519	30.6
117	06Nov2006	3346	62
118	10Nov2006	2200	29.8
119	10Nov2006	2625	29.47
120	14Nov2006	4339	48.7
121	20Nov2006	4738	32.9
122	29Nov2006	1853	35.8
123	29Nov2006	2176	25.6
124	29Nov2006	975	26.1
125	30Nov2006	2087	33.6
126	04Dec2006	1695	34.6
127	05Dec2006	2942	47.9
128	07Dec2006	3260	48.2
129	07Dec2006	4131	47.9
130	11Dec2006	3725	49.8

131	11Dec2006	3460	33.7
132	18Dec2006	1479	33.3
133	21Dec2006	2512	37.2
134	22Dec2006	4077	38.8
135	28Dec2006	1973	49.3
136	29Dec2006	2331	33.4
137	02Jan2007	2359	54.2
138	05Jan2007	2442	25.2
139	09Jan2007	1471	43.7
140	10Jan2007	2727	51.7
141	19Jan2007	2285	43.4
142	24Jan2007	3345	40.2
143	25Jan2007	1537	31
144	30Jan2007	1985	36.3
145	31Jan2007	2835	46.4
146	01Feb2007	2342	41
147	02Feb2007	2910	44.9
148	08Feb2007	4858	58.8
149	12Feb2007	2995	36.6
150	19Feb2007	2087	25.2
151	22Feb2007	3081	39.6
152	19Nov2007	2785	27.7
153	26Nov2007	4411	26
154	26Nov2007	2096	28.6
155	05Dec2007	3116	27.7
156	06Dec2007	1929	39.2
157	06Dec2007	4213	43.7
158	10Dec2007	1652	27.9
159	11Dec2007	3657	49.1
160	27Dec2007	3301	45.8
161	27Dec2007	1958	38.8
162	03Jan2008	3118	29.7
163	04Jan2008	2249	41.6
164	08Jan2008	4926	31.3
165	09Jan2008	1990	28.2
166	11Jan2008	3902	51.5
167	11Jan2008	3621	46.1
168	16Jan2008	2037	31.2
169	17Jan2008	2465	46.7
170	18Jan2008	3168	39.4
171	30Jan2008	2144	44
172	31Jan2008	1293	31.1
173	31Jan2008	2619	40
174	06Feb2008	2170	31.4
175	08Feb2008	2415	52.1

176	14Feb2008	1788	40.5
177	18Feb2008	2288	47.3
178	02Sep2005	6075	44
179	23Sep2005	2283	68
180	30Sep2005	1458	44
181	23Dec2005	1482	31.5
182	25Jan2006	1289	27.1
183	30Jan2006	3103	41.1
184	06Feb2006	1523	45
185	14Mar2006	908	30.5
186	10Apr2006	1923	32.7
187	10Apr2006	2972	45.4
188	12May2006	1304	33.1
189	18May2006	2919	36.7
190	14Jun2006	1504	29.7
191	28Jun2006	2217	51.1
192	03Jul2006	2357	37.8
193	05Jul2006	1387	42.5
194	05Jul2006	2236	36.2
195	07Jul2006	1965	54
196	15Aug2006	1745	28.8
197	23Aug2006	1498	38.3
198	04Sep2006	2172	40.4
199	06Sep2006	1626	36
200	15Sep2006	1237	41
201	20Sep2006	1235	29.5
202	21Sep2006	1551	38.5
203	28Sep2006	1794	45.9
204	03Oct2006	1743	32.2
205	03Oct2006	1453	31.5
206	04Oct2006	1399	28.1
207	11Oct2006	1441	34.7
208	26Oct2006	3150	39.4
209	31Oct2006	2420	55.8
210	09Nov2006	432	27.2
211	10Nov2006	2018	44
212	16Nov2006	1137	37.2
213	17Nov2006	1661	43
214	24Nov2006	1218	26.2
215	05Dec2006	1987	26.8
216	05Dec2006	3011	38.4
217	12Dec2006	1350	32.9
218	03Jan2007	2949	54.2
219	22Jan2007	1410	53.8
220	23Jan2007	918	36.4

221	31Jan2007	3958	43.5
222	05Feb2007	2750	44.6
223	12Feb2007	1845	35.6
224	20Feb2008	2496	35.4
225	26Feb2008	2004	33.2
226	26Feb2008	1997	31
227	05Mar2008	815	33.6

Supplementary Table 3. Immunophenotyping data for subsets of CD4 and CD8 T-cells (manuscript Fig. 5a-c).

Fig. 5a

	CD4:CD8 ratio
**Case**	1.87
Uninfected Children	1.45
	1.37
	1.31
	1.74
	1.59
Uninfected Adults	1.64
	2.00
	1.69
	1.55
	1.81
	0.84
	2.56
	1.62
	1.06
	0.54

Fig. 5b

	Memory T-cell subsets (%)											
	CD4+ T-cells						CD8+ T-cells					
	Naïve	SCM	CM	TM	EM	TE	Naïve	SCM	CM	TM	EM	TE
**Case**	54.9	0.587	21.5	6.33	6.81	0.07	59.2	0.664	5.12	4.13	18.3	5.32
Uninfected Children	61.1	1.28	15.8	10.3	4.2	0.06	42.2	0.444	3.67	11.8	13.9	10.2
	46.5	2.13	24.5	8.07	6.99	1.95	32.1	0.873	2.94	6.83	19	23.6
	64	0.688	19.5	6.27	3.1	0.30	43.8	0.721	3.11	2.1	4.8	22.8
	53.6	2.68	21.3	8.75	4.84	0.03	58.5	2.43	3.74	6.28	9.8	7.31
	64.2	1.87	16.4	5.49	3.86	0.24	51.8	1.04	3.07	6.97	12.6	11.7
Uninfected Adults	41.8	2.87	28.2	5.6	6.4	4.46	49.7	1.03	2.88	5.85	11.9	12.6
	42	2.3	32.6	5.83	5.33	0.07	50.9	0.99	8.85	11.9	18.4	2.21
	41.8	2.39	28.2	10.5	8.82	0.21	40	0.419	5.88	20.1	16.7	5.6
	27.1	2.98	31.9	9.9	13.9	1.81	17	0.911	5.71	10.1	22.4	24.9
	45	5.12	28.5	6.69	4.29	0.32	32.9	1.15	5.9	9.62	14.9	21.5
	27.2	1.8	32.5	6.93	11.3	4.01	14.8	0.318	5.91	15.9	29.9	18.9
	30.9	2.19	47.3	2.95	3.28	0.05	50.1	1.08	8.72	6.44	14.5	5.73
	24.7	1.74	42.4	10	8.21	0.17	6.76	0.431	5.32	8.64	18.9	11.5
	44	2.61	21.9	5.97	9.91	5.75	49.6	1.5	3.05	3.15	6.27	24.9
	9.44	6.1	53	7.43	8.45	0.58	10.6	1.78	7.64	6.41	9.47	39

Fig. 5c

	CCR5, activation and immune-checkpoint markers									
	**CD4+ T-cells**					**CD8+ T-cells**				
	CCR5 density	CCR5%	HLA-DR	TIGIT	PD-1	CCR5 density	CCR5%	HLA-DR	TIGIT	PD-1
**Case**	139.42	11.2	2.18	16.3	17.9	163.43	18.3	5.26	22.6	10.5
Uninfected Children	198.15	10.2	2.86	11	7.89	242.9	21.9	5.04	22.5	2.92
	148.22	14.1	7.93	21	6.69	221.05	17.8	10.6	31.2	3.31
	266.84	10.2	2.45	13.9	6.57	287.16	9.86	2.62	22.9	2.94
	186.64	12.9	3.6	15.5	7.61	250.56	17.9	5.03	17.4	3.47
	124.23	6.13	2.01	12.7	4.35	158.09	14.2	5.16	20	1.68
Uninfected Adults	164.16	4.69	2.45	12.7	7.17	142.02	21.4	4.51	25.6	6.7
	212.22	16	0.97	15.6	7.4	367.92	33.7	1.91	29.4	10.7
	202.22	12.2	1.67	21.4	17.6	231.41	34.7	2.58	41.2	6.32
	147.62	10.3	3.16	19.8	6.18	171.47	44.2	3.87	46.4	6.76
	166.08	10.5	2.36	21.8	9.56	245.83	64.5	*53.3	46	6.67
	190.22	28.4	4.04	22.7	4.57	416.1	38.1	10.1	42.6	8.05
	176.15	8.48	2.59	25.2	10.8	211.03	29.6	10.2	39.4	21.8
	159.89	19.4	0.91	16.6	3.97	282.03	54.3	2.58	62.6	16.3
	170.56	20.4	2.55	22.7	5.93	201.54	36.7	8.97	39.2	11.9
	193.78	14	0.61	26	20.5	212.37	39.7	1.5	59.3	16.7

*Data point (extreme outlier) excluded from Fig. 5c graph – no significant difference between children and adults with or without this data point (two-tailed Mann-Whitney U-test, P>0.05).

Supplementary Table 4. Source of the HIV-1 antigens coupled to carboxylated magnetic beads (Bio-Rad) and the bead regions selected.

HIV-antigen	HIV-1 subtype	Catalogue number	Source	Fluorescence region
gp120	C		Virology, NICD, Prof L Morris	026
gp41	unknown	PR-1204	Jena Bioscience, Germany	034
Vpu-43^{GST}	unknown	PR-380	Jena Bioscience, Germany	043
RT wild type (H66/H51)	unknown	3555	NIH AIDS Reagent Program, Division of AIDS, NIAID, NIH, from Dr Stuart Le Grice and Dr Jennifer T Miller[2]	052
HIV-1 Nef	unknown	11478	NIH AIDS Reagent Program, Division of AIDS, NIAID, NIH	055
HIV-1_{IIIB} pr55 Gag	HIV-1_{IIIB} strain	3276	NIH AIDS Reagent Program, Division of AIDS, NIAID, NIH	062
V1V2 scaffold	C (CAP257)		Virology, NICD, Prof L Morris	063
Vpu 9	C (STMVDMGHLR LLDVNDL)		NMI Natural and Medical Sciences Institute, University Tuebingen, Germany	064
HIV-1_{IIIB} Tat	HIV-1_{IIIB} strain	2222	NIH AIDS Reagent Program, Division of AIDS, NIAID, NIH[3]	065
HIV immunoglobulin (Positive control)	Asymptomatic HIV antibody positive donors	3957	NIH AIDS Reagent Program, Division of AIDS, NIAID, NIH, from NABI and National Heart Lung and Blood Institute (Dr Luiz Barbosa)	

Supplementary Table 5. PCR primer sequences used for the real-time AS-PCR *KIR* genotyping assay.

Well	*KIR* gene target	Primer sequence (5'-3')	*KIR* alleles not detected
1	[a]2DL1 f1	GTTGGTCAGATGTCATGTTTGAA	*013N
	[a]2DL1 r1	GGTCCCTGCCAGGTCTTGCG	
2	[a]2DL1 f2	TGGACCAAGAGTCTGCAGGA	*005
	[a]2DL1 r2	TGTTGTCTCCCTAGAAGACG	*0040102
3	[a]2DL2 f1	CTGGCCCACCCAGGTCG	*004;*00601;*00602
	[a]2DL2 r1	GGACCGATGGAGAAGTTGGCT	*00303;*004
4	[b]2DL2 f2	AAACCTTCTCTCTCAGCCCA	
	[b]2DL2 r2	GCCCTGCAGAGAACCTACA	*009
5	[b] 2DL3 f1	AGACCCTCAGGAGGTGA	
	[b] 2DL3 r1	CAGGAGACAACTTTGGATCA	*010;*017
6	[d]2DL3 f2	GGCCCAGAAGTGCCCTCT	
	[d]2DL3 r2	ATTGGAGCTGGCAACCCA	
7	[a] 2DL4 f1	CAGGACAAGCCCTTCTGC	
	[a]2DL4 r1	CTGGGTGCCGACCACT	
8	[a]2DL4 f2	ACCTTCGCTTACAGCCCG	
	[a]2DL4 r2	CCTCACCTGTGACAGAAACAG	
9	[a]2DL5 f1	GCGCTGTGGTGCCTCG	
	[a]2DL5 r1	GACCACTCAATGGGGGAGC	
10	[a]2DL5 f2	TGCAGCTCCAGGAGCTCA	
	[a]2DL5 r2	GGGTCTGACCACTCATAGGGT	
11	[b]2DS1 f1a	TCTCCATCAGTCGCATGAG	
	[b]2DS1 f1b	TCTCCATCAGTCGCATGAA	
	[b]2DS1 r1	GGTCACTGGGAGCTGAC	
12	[d]2DS1 f2	CCGCTCTTGAGCGAGCAA	
	[d]2DS1 r2	CAACTCCACCTCCAGGCCTATATA	
13	[a]2DS2 f1	TTCTGCACAGAGAGGGGAAGTA	
	[a]2DS2 r1	GGGTCACTGGGAGCTGACAA	
14	[a] 2DS2 f2	CGGGCCCCACGGTTT	*00104
	[a] 2DS2 r2	GGTCACTCGAGTTTGACCACTCA	
15	[c]2DS3 f1	AAACCTTCTCTCTCAGCCCA	
	[c]2DS3 r1	GCATCTGTAGGTTCCTCCT	
16	[a] 2DS3 f2	CTATGACATGTACCATCTATCCAC	

	[a] 2DS3 r2	AAGCAGTGGGTCACTTGAC	
17	[a] 2DS4 f1	CTGGCCCTCCCAGGTCA	
	[a] 2DS4 r1	TCTGTAGGTTCCTGCAAGGACAG	
18	[a] 2DS4 f2	GTTCAGGCAGGAGAGAAT	
	[a] 2DS4 r2	GTTTGACCACTCGTAGGGAGC	
19	[a]2DS5 f1	TGATGGGGTCTCCAAGGG	
	[a]2DS5 r1	TCCAGAGGGTCACTGGGC	*003
20	[a]2DS5 f2	ACAGAGAGGGGACGTTTAACC	
	[a]2DS5 r2	ATGTCCAGAGGGTCACTGGG	
21	[a]2DP1 f1	GTCTGCCTGGCCCAGCT	
	[a]2DP1 r1	GTGTGAACCCCGACATCTGTAC	
22	[a]2DP1 f2	CCATCGGTCCCATGATGG	
	[a]2DP1 r2	CACTGGGAGCTGACAACTGATG	
23	[a]3DL1 f1	CGCTGTGGTGCCTCGA	*009;*042;*057
	[a]3DL1 r1	GGTGTGAACCCCGACATG	
24	[b]3DL1 f1a	CCATCGGTCCCATGATGCT	*054
	[b]3DL1 f1b	CCATTGGTCCCATGATGCT	*054
	[b]3DL1 f1c	TCCATCGGTCCCATGATGTT	*054
	[b]3DL1 r2	CCACGATGTCCAGGGGA	
25	[a]3DL2 f1	CAAACCCTTCCTGTCTGCCC	*01301;*01302;*014;*024;*025;<br>*037;*040;*046;*052;*054
	[a]3DL2 r1	GTGCCGACCACCCAGTGA	
26	[a]3DL2 f2	CCCATGAACGTAGGCTCCG	
	[a]3DL2 r2	CACACGCAGGGCAGGG	*018
27	[a]3DL3 f1	GTCAGGACAAGCCCTTCCTC	
	[a]3DL3 r1	GAGTGTGGGTGTGAACTGCA	
28	[a]3DL3 f2	TTCTGCACAGAGAGGGGATCA	
	[a]3DL3 r2	GAGCCGACAACTCATAGGGTA	
29	[a]3DS1 f1	AGCCTGCAGGGAACAGAAG	
	[a]3DS1 r1	GCCTGACTGTGGTGCTCG	
30	[b]3DS1f 2a	CATCGGTTCCATGATGCG	
	[b]3DS1f 2b	CATCAGTTCCATGATGCG	
	[b]3DS1 r2	CCACGATGTCCAGGGGA	
31	[b]3DP1 f1	GTACGTCACCCTCCCATGATGTA	*00902;*004
	[d]3DP1 r1	GCACTCCCTCCCTCTATTCCT	
32	[d]3DP1 f2	CATCCTCCTCTCTAAGGTGGC	
	[b]3DP1 r2	GAAAACGGTGTTTCGGAATAC	

All	[c]GALC f	TTACCCAGAGCCCTATCGTTCT
	[c]GALC r	GTCTGCCCATCACCACCTATT

GALC, galactosylceramidase; KIR, killer-cell immunoglobulin-like receptor; PCR, polymerase chain reaction; AS-PCR, allele-specific PCR; Primers taken from [a]Martin and Carrington[4], [b]Vilches *et al.*[5], [c]Alves *et al.*[6], and [d]Gentle *et al*.[7]

Supplementary Table 6. List of PCR primers and probe sequences used for real-time quantitative AS-PCR copy number determination of KIR3DL1/S1 and KIR2DS4f/v.

Well	Primer name	Primer sequence (5'-3')
1	*KIR3DL1* f1[a]	GCCTCGTTGGACAGATCCA[T]
	KIR3DL1 r1[a]	TAGGTCCCTGCAAGGGCA[A]
	KIR3DL1/S1 probe[a]	VIC-GGGTCTCCAAGGCCAATTTCTCCAT-MGB
2	*KIR3DS1* f1[a]	CTCGTTGGACAGATCCATG[A]
	KIR3DS1 r1[a]	GTCCCTGCAAGGGCA[C]
	KIR3DL1/S1 probe[a]	VIC-GGGTCTCCAAGGCCAATTTCTCCAT-MGB
3	*KIR2DS4f* f1[b]	CCGGAGCTCCTATGACATG
	KIR2DS4f/v r1[b]	TGACGGAAACAAGCAGTGG[A]
	KIR2DS4f/v probe[b]	VIC-AACATTCCAGGCCGACTTTCCTCTG-MGB
4	*KIR2DS4v* f1[b]	CCTTGTCCTGCAGCTCCAT
	KIR2DS4f/v r1[b]	TGACGGAAACAAGCAGTGG[A]
	KIR2DS4f/v probe[2]	VIC-AACATTCCAGGCCGACTTTCCTCTG-MGB
All	*BGB* f1[c]	TCGCTTTCTTGCTGTCCAATTTCTA
	BGB r1[c]	ATGCTCAAGGCCCTTCATAATATCC
	BGB probe[c]	FAM-CCTAAGTCCAACTACTAAACTG-MGB

VIC and FAM, probe reporter fluorescent dyes; MGB, minor groove binding protein (non-fluorescent quencher); AS-PCR, allele-specific PCR; *BGB*, human beta-globin, [ ] denotes 3' locked nucleic acid (LNA) base modification. [a]Primers/probes taken from Pelak *et al*.[8], [b]Jiang *et al.*[9], and [c]Shostakovich-Koretskaya *et al*. [10]

Supplementary References

1 Lugli, E. *et al.* Identification, isolation and in vitro expansion of human and nonhuman primate T stem cell memory cells. *Nat. Protoc.* **8**, 33-42 (2013).

2 Le Grice, S. F., Cameron, C. E. & Benkovic, S. J. Purification and characterization of human immunodeficiency virus type 1 reverse transcriptase. *Methods Enzymol.* **262**, 130-144 (1995).

3 Bohan, C. A. *et al.* Analysis of Tat transactivation of human immunodeficiency virus transcription in vitro. *Gene Expr.* **2**, 391-407 (1992).

4 Martin, M. P. & Carrington, M. KIR locus polymorphisms: genotyping and disease association analysis. *Methods Mol. Biol.* **415**, 49-64 (2008).

5 Vilches, C., Castano, J., Gomez-Lozano, N. & Estefania, E. Facilitation of KIR genotyping by a PCR-SSP method that amplifies short DNA fragments. *Tissue Antigens* **70**, 415-422 (2007).

6 Alves, L. G., Rajalingam, R. & Canavez, F. A novel real-time PCR method for KIR genotyping. *Tissue Antigens* **73**, 188-191 (2009).

7 Gentle, N. L., Loubser, S., Paximadis, M., Puren, A. & Tiemessen, C. T. Killer-cell immunoglobulin-like receptor (KIR) and human leukocyte antigen (HLA) class I genetic diversity in four South African populations. *Hum. Immunol.* **78**, 503-509 (2017).

8 Pelak, K. *et al.* Copy number variation of KIR genes influences HIV-1 control. *PLoS Biol.* **9**, e1001208 (2011).

9 Jiang, W. *et al.* Copy number variation leads to considerable diversity for B but not A haplotypes of the human KIR genes encoding NK cell receptors. *Genome Res.* **22**, 1845-1854 (2012).

10 Shostakovich-Koretskaya, L. *et al.* Combinatorial content of CCL3L and CCL4L gene copy numbers influence HIV-AIDS susceptibility in Ukrainian children. *AIDS* **23**, 679-688 (2009).
