# Supplementary material for: A child with perinatal HIV infection and long-term sustained virological control following antiretroviral treatment cessation
Source: Nat Commun. 2019 Jan 24;10:412. doi: 10.1038/s41467-019-08311-0 (PMC6345921; doi:10.1038/s41467-019-08311-0)
Supplement: Supplementary file 3 — Reporting Summary [file 41467_2019_8311_MOESM3_ESM.pdf]

nature research

Corresponding author(s): Caroline T. Tiemessen, Avy Violari

Last updated by author(s): Jan 3, 2019

Reporting Summary

Nature Research wishes to improve the reproducibility of the work that we publish. This form provides structure for consistency and transparency in reporting. For further information on Nature Research policies, see Authors & Referees and the Editorial Policy Checklist.

Statistics

For all statistical analyses, confirm that the following items are present in the figure legend, table legend, main text, or Methods section.

n/a	Confirmed	
☐	☒	The exact sample size (*n*) for each experimental group/condition, given as a discrete number and unit of measurement
☐	☒	A statement on whether measurements were taken from distinct samples or whether the same sample was measured repeatedly
☐	☒	The statistical test(s) used AND whether they are one- or two-sided<br>*Only common tests should be described solely by name; describe more complex techniques in the Methods section.*
☒	☐	A description of all covariates tested
☒	☐	A description of any assumptions or corrections, such as tests of normality and adjustment for multiple comparisons
☐	☒	A full description of the statistical parameters including central tendency (e.g. means) or other basic estimates (e.g. regression coefficient) AND variation (e.g. standard deviation) or associated estimates of uncertainty (e.g. confidence intervals)
☒	☐	For null hypothesis testing, the test statistic (e.g. *F*, *t*, *r*) with confidence intervals, effect sizes, degrees of freedom and *P* value noted<br>*Give P values as exact values whenever suitable.*
☒	☐	For Bayesian analysis, information on the choice of priors and Markov chain Monte Carlo settings
☒	☐	For hierarchical and complex designs, identification of the appropriate level for tests and full reporting of outcomes
☒	☐	Estimates of effect sizes (e.g. Cohen's *d*, Pearson's *r*), indicating how they were calculated

Our web collection on statistics for biologists contains articles on many of the points above.

Software and code

Policy information about availability of computer code

Data collection	Excel 2016, Microsoft Office for Case study and controls; DataFax version 3.8.0 was used to collect data for the CHER study.
Data analysis	GraphPad Prism version 5.0 for Case study and controls; SAS 9.3 and SAS Enterprise Guide was used to analyse the CHER data.

For manuscripts utilizing custom algorithms or software that are central to the research but not yet described in published literature, software must be made available to editors/reviewers. We strongly encourage code deposition in a community repository (e.g. GitHub). See the Nature Research guidelines for submitting code & software for further information.

Data

Policy information about availability of data

All manuscripts must include a data availability statement. This statement should provide the following information, where applicable:

- Accession codes, unique identifiers, or web links for publicly available datasets
- A list of figures that have associated raw data
- A description of any restrictions on data availability

The data that support the findings in this study are available from the corresponding authors upon reasonable request. The source data underlying Figs 1a-c and 5a-c are provided in Supplementary Tables 1 and 3, respectively. Genbank accession numbers for cloned gag proviral sequences: MH789553-MH789572 [https://www.ncbi.nlm.nih.gov/nucleotide/].

Field-specific reporting

Please select the one below that is the best fit for your research. If you are not sure, read the appropriate sections before making your selection.

☒ Life sciences ☐ Behavioural & social sciences ☐ Ecological, evolutionary & environmental sciences

For a reference copy of the document with all sections, see nature.com/documents/nr-reporting-summary-flat.pdf

Life sciences study design

All studies must disclose on these points even when the disclosure is negative.

Sample size	Uninfected children of similar age to the child case were recruited (n=5) to allow comparison with uninfected adults (n=10). These served as comparators for the child's single data point and to assess whether 10 year old children have adult-like expression of the various cell markers. This number of participants was chosen to provide descriptive results.
Data exclusions	Flow cytometry immunophenotyping: one adult outlier (extreme) data point was omitted from the CD8+HLA-DR+ graph (value given and indicated in Supplementary Table 3); the adult vs children comparison was not statistically different with or without this data point (two-tailed Mann Whitney U-test; $P > 0.05$).
Replication	Assays to detect HIV-1 DNA or RNA - as many replicates were conducted as the sample availability would allow for a particular time point. Replicates for molecular tests were conducted to maximize sensitivity of detection of a small reservoir i.e. the greater the input of nucleic acid the greater the number of CD4 T-cells being tested. Detection of viral RNA plasma by standard VL tests (in larger volume of 10 ml) at >9.5 years of age was confirmed using an in-house nested PCR with inclusion of a DNAse treatment step to ensure no contaminating cellular DNA accounts for this measure (which is not a step included in standard diagnostic VL tests).<br>Viral outgrowth assays (measuring ability to reactivate virus) and testing permissiveness of child's CD4 T-cells to infection with HIV-1 BaL, included the maximum number of cells available at the particular time points, and included the use of two different assays for measuring viral induction or permissiveness (MOLT4/CCR5 and primary CD4 T-cells from a high expressing CCR5 donor) at 9.5 years of age (only primary cells were used at 50 weeks of age due to sample limitation and for virus reactivation experiments only).<br>Stringent criteria were used for determining a positive HIV-specific antibody result in the multiplex bead assay (mean of control data +/- 3 SD); indeterminate Western blot result verifies a response to Gag. The intracellular cytokine assay was conducted once at 9.5 years of age.<br>Immunophenotyping by flow cytometry and host genotyping were conducted using 9.5 year whole blood sample - conducted once.
Randomization	There was no randomization and no experimental intervention group. As this is a case study, other participants were used to provide context and descriptive results. For the flow cytometry component - children were matched for age to the child (3 females, 2 males). Adults (5 females, 5 males) and children were all from the same ethnic population as the child (black South African).
Blinding	Investigators were not blinded during data collection or analysis. The investigators who conducted the flow cytometry component (assays and analysis) recruited the uninfected control patients, as samples were whole blood samples that required immediate processing for flow cytometry they had knowledge of which sample belonged to a specific group. The CHER trial, in which this child case was originally enrolled, was an open label randomized trial.

Reporting for specific materials, systems and methods

We require information from authors about some types of materials, experimental systems and methods used in many studies. Here, indicate whether each material, system or method listed is relevant to your study. If you are not sure if a list item applies to your research, read the appropriate section before selecting a response.

Materials & experimental systems

n/a	Involved in the study	
☐	☒	Antibodies
☐	☒	Eukaryotic cell lines
☒	☐	Palaeontology
☒	☐	Animals and other organisms
☐	☒	Human research participants
☐	☒	Clinical data

Methods

n/a	Involved in the study	
☒	☐	ChIP-seq
☐	☒	Flow cytometry
☒	☐	MRI-based neuroimaging

Antibodies

Antibodies used	Quantitation of HIV specific antibody isotypes/subclasses: R-phycoerthyrin-conjugated mouse anti-human IgG1 to IgG4 (Cat. No. 9052-09, 9070-09, 9210-09, 9200-09, respectively, Southern Biotech, USA), mouse anti-human IgM (Cat. No. 9020-09, Southern Biotech), mouse anti-human IgA1 (Cat. No.9130-09, Southern Biotech) or mouse anti-human IgA2 (Cat. No. 9140-09, Southern Biotech).<br>Intracellular cytokine assay: costimulatory antibodies CD28 and CD49d (Cat. No. 340975 and 340976, respectively, BD BioSciences). Anti-CD16 antibody (Clone eBioCB16, Cat. No. 16-0168-85, eBioscience, San Diego, California, US). CD3 PerCP

October 2018

(clone SK7, Cat. No.345766), CD8 FITC (clone SK1, Cat. No. 347313,), CD4 BV786 (clone L200, Cat. No. 563914), CD56 APC (clone NCAM16.2, Cat. No. 341025), IL-2 PE (clone 5344.111, Cat. No. 340450) and IFN γ PE (clone 25723.11, Cat. No. 340452). Immunophenotyping by flow cytometry: CD3 APC-H7 (clone SK7, Cat. No. 560176), CD8 PerCP (clone SK1, Cat. No.347314), CD8 Alexa Fluor 700 (clone RPA-T8, Cat. No. 557945), CD4 BV786 (clone L200, Cat. No. 563914), CD4 FITC (clone SK3, Cat. No. 347413), CCR5 PE (clone 2D7, custom 1:1 conjugated antibody), CCR7 FITC (clone 150503, Cat. No. 150503), CD45RO BV510 (clone UCHL1, Cat. No. 563215), CD62L PE-CF594 (clone DREG-56), PD-1 BV786 (clone EH12.1) - all from BD Biosciences. TIGIT APC (MBSA43, Cat. No. 562301) - eBioscience, HLA-DR PE-Cy5.5 (clone TU36, Cat. No. MHLDR18) - Life technologies and CD95 BV605 (clone DX2, Cat. No. 305628) - Biolegend (San Diego, California, USA). The CCR5 antibody was conjugated to PE at a ratio of 1:1, and quantitation (density measured as antibodies bound per cell) carried out using the QuantiBRITE system (BD Biosciences).

Validation	Antibodies (Abs) used were developed for the specific applications, and QC testing was done on human samples by the suppliers. Optimal Ab concentrations for use were determined by titration, and empirical testing of Ab combinations was done to establish the individual flow cytometry panels.

Eukaryotic cell lines

Policy information about cell lines

Cell line source(s)	MOLT4/CCR5 cells (Cat. No. 4984) - obtained from the NIH AIDS Reagent Program, NAIAD, NIH from Dr. Masanori Baba, Dr. Hiroshi Miyake, Dr. Yuji Iizawa.
Authentication	CCR5 expression on MOLT4/CCR5 cells was verified in-house by flow cytometry; used as positive controls to assess cell permissiveness to virus infection.
Mycoplasma contamination	These were purchased mycoplasma, bacteria and fungi free; a new vial of cells was received and used. No in-house testing for mycoplasma contamination was done in addition.
Commonly misidentified lines (See ICLAC register)	No commonly misidentified cell line was used in this study.

Human research participants

Policy information about studies involving human research participants

Population characteristics	One HIV-1 infected male participant age 9.5 years (the case); 5 HIV uninfected children (3 females, 2 males) median age 9 years; 10 uninfected adults (5 females, 5 males) median age 44 years.
Recruitment	The child case was recruited from prevention of mother to child transmission programmes in the greater Johannesburg area along with 450 other children who participated in CHER. The case was randomized to early treatment.
Ethics oversight	The Ethics Committees of the University of the Witwatersrand and Stellenbosch University (CHER trial). The Human Research Ethics Committee of the University of the Witwatersrand provided approval for all subsequent observational studies and investigations of the Case.

Note that full information on the approval of the study protocol must also be provided in the manuscript.

Clinical data

Policy information about clinical studies

All manuscripts should comply with the ICMJE guidelines for publication of clinical research and a completed CONSORT checklist must be included with all submissions.

Clinical trial registration	The study reported in the manuscript is not a clinical trial - the child case being studied was originally recruited as part of the CHER clinical trial. The CHER trial (ClinicalTrials.gov Identifier: NCT00102960).
Study protocol	The full protocol of the CHER study cannot be accessed as the CIPRA website is now closed. A synopsis of the protocol and study results can be found under ClinicalTrials.gov. The protocol can be requested from the corresponding authors.
Data collection	Recruitment into the CHER trial was between 2005-2011, in Stellenbosch and Soweto, South Africa; subsequent to this further follow up and data collection on the child case is ongoing as part of the child's care and approved observational studies.
Outcomes	There are no pre-defined primary or secondary outcome measures for this study as it is a descriptive study. The primary outcome for the CHER study was time to death or failure of first line therapy. Clinical evaluations, CD4 and viral load measurements were used to assess the primary outcomes.

Flow Cytometry

Plots

Confirm that:

☒ The axis labels state the marker and fluorochrome used (e.g. CD4-FITC).

☒ The axis scales are clearly visible. Include numbers along axes only for bottom left plot of group (a 'group' is an analysis of identical markers).

☒ All plots are contour plots with outliers or pseudocolor plots.

☒ A numerical value for number of cells or percentage (with statistics) is provided.

Methodology

Sample preparation	Whole blood was stained using 3 different antibody staining panels. Whole blood (100 ul) was incubated with the antibodies at room temperature, in the dark, for 15 minutes. Thereafter, red blood cells were lysed using FACS lysing solution (BD Biosciences). Samples were then washed and resuspended in FACSflow, and acquired on the flow cytometer.
Instrument	A 4-laser BD LSRFortessaTM X-20 flow cytometer (BD Biosciences).
Software	FlowJo Software (Tree Star Inc., Ashland, OR).
Cell population abundance	Immunophenotyping was conducted using whole blood samples (100 ul per staining panel); no post-sorting of cells was involved.
Gating strategy	Singlets were identified followed by gating on CD3+ lymphocytes. CD3+ T-cells were further gated on low SSC-A and FSC-A (small lymphocytes) and then on distinct populations of CD4+ and CD8+ T-cells, and then on select combinations of markers identifying specific cell subsets - using 3 staining panels. For the low frequency SCM (stem cell memory) subset, the gating strategy outlined by Lugli et al, Nature Protocols 8, 33-42 (2013) was utilized: the slightly lower level of CCR7 expression on SCM compared to Naive T-cells was utilized to provide a clear separation of positive and negative CD95 cells. A diagonal gate was drawn on the bivariate plot of CD95 versus CCR7 on the total T cell population and then copied into the same bivariate plot after gating for CD45RO, CCR7 and CD62L.

☒ Tick this box to confirm that a figure exemplifying the gating strategy is provided in the Supplementary Information.
